# Eco-evo-devo of the lemur syndrome: did adaptive behavioral plasticity get canalized in a large primate radiation?

**DOI:** 10.1186/1742-9994-12-S1-S15

**Published:** 2015-08-24

**Authors:** Peter M Kappeler, Claudia Fichtel

**Affiliations:** 1 Behavioral Ecology and Sociobiology Unit, German Primate Center, Kellnerweg 4, 37077 Göttingen, Germany

**Keywords:** evolution, behavioral development, masculinization, canalization, lemurs

## Abstract

**Background:**

Comprehensive explanations of behavioral adaptations rarely invoke all levels famously admonished by Niko Tinbergen. The role of developmental processes and plasticity, in particular, has often been neglected. In this paper, we combine ecological, physiological and developmental perspectives in developing a hypothesis to account for the evolution of ‘the lemur syndrome’, a combination of reduced sexual dimorphism, even adult sex ratios, female dominance and mild genital masculinization characterizing group-living species in two families of Malagasy primates.

**Results:**

We review the different components of the lemur syndrome and compare it with similar adaptations reported for other mammals. We find support for the assertion that the lemur syndrome represents a unique set of integrated behavioral, demographic and morphological traits. We combine existing hypotheses about underlying adaptive function and proximate causation by adding a potential developmental mechanism linking maternal stress and filial masculinization, and outline an evolutionary scenario for its canalization.

**Conclusions:**

We propose a new hypothesis linking ecological, physiological, developmental and evolutionary processes to adumbrate a comprehensive explanation for the evolution of the lemur syndrome, whose assumptions and predictions can guide diverse future research on lemurs. This hypothesis should also encourage students of other behavioral phenomena to consider the potential role of developmental plasticity in evolutionary innovation.

## 

“… natural selective factors impinge not on the hereditary factors themselves, but on the organisms as they develop from fertilized eggs to reproductive adults. We need to bring into the picture not only the genetic system by which hereditary information is passed from one generation to the next, but also the “epigenetic system” by which the information contained in the fertilized egg is expanded into the functioning structure of the reproducing individual. Each organism during its lifetime will respond in some manner to the environmental stresses to which it is submitted, and in a population there is almost certain to be some genetic variation in the intensity and character of these responses. Natural selection will favour those individuals in which the responses are of most adaptive value.”

C. H. Waddington (1959) p. 1635

## Background

Comprehensive studies of behavior ideally include multiple perspectives, including not only investigations of adaptive function, but also of underlying mechanisms, developmental processes and the phylogenetic history of a trait [1]. Historically, behavioral ecologists have primarily focused on the function of a behavior in light of environmental factors that improve optimization of this trait [2]. By also studying the developmental causes of behavioral variation, patterns of variation among closely related species and their ancestors, and how this variation is brought about by neuro-endocrine mechanisms, today's behavioral biologists are increasingly paying more attention to the perspectives that characterized much work by classical ethologists [3,4]. These more comprehensive approaches increasingly reveal details about the processes and mechanism underlying both, the flexibility of behavior and the constraints imposed upon it [5,6].

Behavior varies across several spatio-temporal scales, including over the lifetime of an individual, among individuals within social units or populations, among populations of a species and among closely related species. Identification of the forces and mechanisms that produce variability at these different levels, but also of those that conserve adaptive behavioral solutions, is a major goal in behavioral biology (reviewed in [5,7-10]). Studies of consistent individual variation in various behavioral traits, many of which are correlated across time and contexts, provide opportunity for identifying the factors and processes that generate both, behavioral diversity and uniformity [11-14]. The fact that individuals under virtually identical social and ecological conditions can and do express different stable behavior patterns indicates substantial genetic influences on these traits [8,15]. Significant genetic influences on such personality traits have indeed been documented, and some are correlated with morphological and life-history traits that affect individual fitness [15,16]. Thus, we are beginning to understand what part of phenotypic variation in a behavioral trait can be attributed to additive genetic variation, epistatic, maternal or environmental effects [17,18], but phenotype shaping through epigenetic or other developmental processes may also contribute to inter-individual variation [19-23].

Little is still known about the interaction between developmental and evolutionary processes in shaping phenotypic variation in behavioral traits, however. While integration of evolutionary biology with developmental genetics into ‘evo-devo’ afforded a deeper understanding of multicellular development and morphological evolution (e.g. [24]), this approach has only rarely been applied to behavioral traits. However, it has, for example, been proposed that molecular pathways controlling feeding behavior and reproduction in solitary insects are part of a genetic toolkit underlying the evolution of the division of labor in honeybee workers [25]. An even more comprehensive approach posited that environmental factors generate and induce genotypic and phenotypic variation at multiple levels of biological organization, while development acts as a regulator that can mask, release, or create new combinations of variation [26]. If natural selection subsequently fixes this variation, novel stable phenotypes can emerge [20]. Because behavioral phenotypes respond much more flexibly to environmental factors than other traits [5], such an ‘eco-evo-devo approach’ may also improve our understanding of other behavioral phenomena. Here, we develop the hypothesis that a suite of behavioral and morphological traits characterizing virtually an entire primate radiation may represent an example of adaptive canalization of a developmental process, a general mechanism first suggested by Waddington [19] that has only recently received renewed attention [26-28].

## The lemur syndrome

The living primates of Madagascar (Lemuriformes) represent the endpoints of an adaptive radiation following a single colonization in the Eocene [29]. The more than 100 species from 5 families, and at least 17 species that went extinct in recent centuries following human colonization of the island [30], exhibit diversity in life history, ecology and social systems that rivals that of all other primates combined [31-34]. In colonizing different habitats, ranging from arid spiny forest to humid rain forest, lemurs have diversified into the smallest (30g) as well as some of the largest primate species (160 kg) with corresponding fast and slow life histories, diverse activity patterns, dietary specializations and types of social organization [34]. About 20% of the living lemur species are group living, i.e. their bisexual groups contain on average at least 3 adults. The currently available phylogenetic evidence suggests that group living evolved twice independently; once in the Lemuridae (genera *Lemur, Eulemur, Hapalemur* and *Varecia*) and once in the Indriidae (genus *Propithecus*) [35,36].

Group-living lemurs exhibit a combination of morphological and social traits that have been referred to as the lemur syndrome [37], and which contrasts strongly with patterns found among other primates as well as most other mammals (see below). The co-occurrence of the main components of the lemur syndrome (lack of sexual dimorphism, even adult sex ratios, female dominance and mild genital masculinization) indicates both, a potential functional and or proximate connection as well as apparent deviations from predictions of sexual selection and socio-ecological theory. In this section, we will first review the components of the lemur syndrome and contrast it with similar patterns in other mammals before reviewing existing hypotheses about its origin and function. We will then present a hypothesis and a mechanism that combine ecological, developmental and evolutionary processes to account for the evolution of the lemur syndrome.

### Morphology

Sexual selection theory predicts that intrasexual selection will lead to sex differences in morphological traits if those traits confer a competitive advantage. In mammals, sex differences in body size and in the presence or size of species-specific weapons, such as antlers, horns, or canines therefore vary as a function of the mating system. Specifically, promiscuous and especially polygynous species exhibit more pronounced sexual dimorphism in body and weapon size than species with monogamous and polyandrous mating systems, which are characterized by reduced variance in male reproductive success [38,39]. Patterns of sexual dimorphism can also be shaped by selection on females, however. Non-sexual social selection [40], which evaluates differential reproductive success arising from competition for resources other than mates, appears common among vertebrates [41], but high variance in female reproductive success is rarely accompanied by female-biased sexual dimorphism, at least among mammals. In cooperatively breeding mammals, for example, interactions with kin selection may render variance in female inclusive fitness lower than predicted, making conflict resolution among females with overt aggression less important, which in turn reduces selection on determinants of competitive ability [42]. Because only a minority of mammals are cooperative breeders [43], the relative importance of sex-specific determinants of mammalian sexual dimorphism remain poorly understood, however (see e.g. [44]).

Comparative studies of mammalian mating systems are still hampered by a lack of information on mating patterns, especially in nocturnal or secretive species, and the reproductive system, i.e. who fertilized the eggs; the latter requiring genetic paternity analyses. Most comparative studies have therefore relied on more readily available information on social organization, i.e. the size and composition of social units. Accordingly, mammals living in pairs are commonly considered as monogamous and contrasted with solitary and group-living species with polygamous mating systems [45,46].

**Figure 1:**
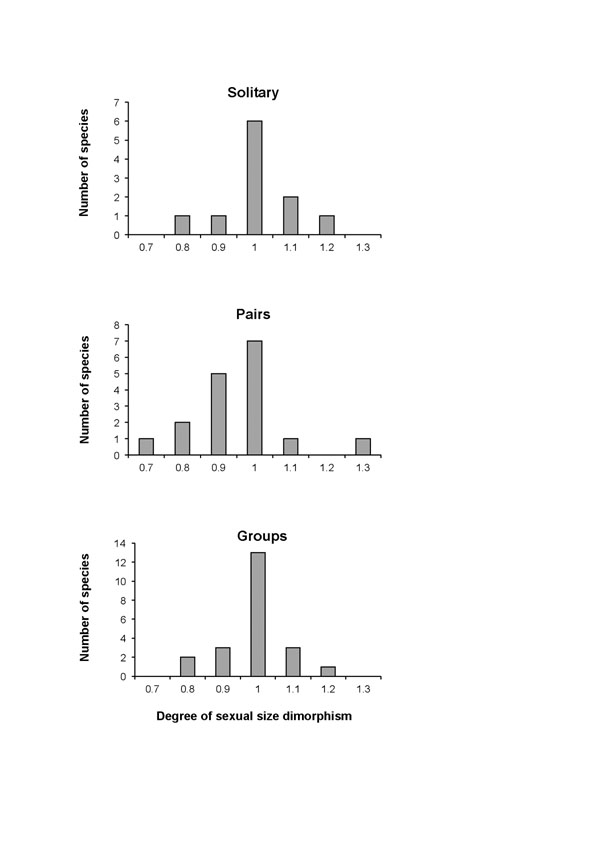
Histograms of the number of lemur species with different social organizations exhibiting different degrees of sexual size dimorphism. The ratios of male/female mean adult body mass have been combined into several categories. For example, “1” represents a category where the degree of sexual size dimorphism varies between 0.95 and 1.05. Body mass data were taken from [43,199]. Definitions of social organization follow [212].

Because genetic paternity studies have been conducted for only a handful of lemur species, their classification for comparative studies has been based on data on social organization, which is available for about half of all lemur species. Analysis of a recent summary of lemur body mass data (based on [47]) confirms results of earlier studies [48-50]: all lemur species, irrespective of their social organization, lack significant sexual size dimorphism (Figure 1). Variation in individual data sets due to different living conditions (captive vs. wild), age classifications or seasonal variation does not obscure the fact that there is no lemur species in which males are significantly larger than females, even though male-male competition during the short annual breeding season characterizing most lemur species has been amply demonstrated [51-54]. A similar picture emerged from a comparative analysis of sexual dimorphism in the size of canines, which represent the most important weapons of primates [55,56]. Moreover, none of the extinct large sub fossil lemurs, where more pronounced sexual dimorphism is expected based on allometric effects [57], exhibited any significant sexual dimorphism [58]. Finally, female lemurs are not only remarkable morphologically because of their relatively large body size compared to males. They also have moderately masculinized genitals, characterized by a slightly enlarged pendulous external clitoris [59,60].

The absence of male-biased sexual dimorphism in polygamous lemurs is remarkable for two reasons. First, although polyandrous mating and the ensuing sperm competition are common in some species, which is reflected by above-average relative testes size, adaptations to intense sperm competition are not so common across group-living lemurs as to provide a unifying explanation for the lack of sexual dimorphism in the entire clade [61]. Second, low levels of male reproductive skew could theoretically explain the lack of sexual dimorphism as the result of some as yet unknown mechanism of post-copulatory selection. However, male reproductive skew appears generally high; in one sexually monomorphic lemur species (*Propithecus verreauxi*) even exceeding the extent of reproductive skew observed in highly sexually-dimorphic gorillas [37,54,62]. Thus, the lack of male-biased sexual dimorphism is widespread among polygamous lemurs, in contrast to patterns in other primates and mammals [63-65], and clearly not expected based on mechanisms and predicted consequences of pre- and post-copulatory competition among males. To what extent social selection on females or environmental factors may affect patterns of sexual dimorphism will be discussed below.

### Social organization

Even birth sex ratios are evolutionarily stable in most vertebrates. Subsequent juvenile and adult mortality typically lead to deviations from even sex ratios because early mortality in most species is sex-biased. In primates and other mammals, where potential reproductive rates of males exceed those of females, adaptations to prevail in pre-copulatory mating competition are associated with physiological costs for males that, together with elevated risks of injury, infection, disease and ultimately death, will result in male-biased mortality [66]. As a result, adult sex ratios in polygamous mammals are typically female-biased, whereas they remain even in monogamous species [67].

The intensity of intrasexual selection is closely associated with male monopolization potential, i.e. the ability of individual males to exclude rivals from potential mates. Male monopolization potential is determined by independent variation in the number of spatially clumped females and the temporal distribution of their receptive periods [68]. In anthropoid primates, female group sizes of about 6 are conducive to monopolization by a single male; groups with more adult females also contain multiple males [69]. In group-living lemurs, in contrast, average adult sex ratios (ASR) are equal, or even male-biased, even though the largest groups contain on average only 5 adult females [70-72] (see also [73] for a pair-living species). This failure of single males to monopolize groups is not due to strong female receptive synchrony, however, because the receptive periods of individual females are so spread out in time that they are effectively asynchronous, despite pronounced seasonality of mating [74,75]. The adult sex ratios of solitary lemurs are less well documented, but there is also no apparent evidence for the expected female-bias [76-79].

Analysis of the unexpected adult sex ratios in group-living lemurs touches upon three central questions in behavioural ecology. First, there is an interesting conflict of interest between dominant and subordinate males over group membership [80]. In *P. verreauxi*, dominants do not appear to benefit from subordinate males joining their group in terms of reduced take-over risk or improved group productivity [81,82], whereas in *Eulemur rufifrons *communal male territorial defence appears to outweigh the dominants’ cost of sharing reproduction [83]. Thus, it is not yet generally understood why dominant resident males should accept immigration of additional males, but intruder pressure, which is ultimately related to population density and fecundity, may be unusually high [80]. Second, there might be conflict between the sexes over group composition, but this aspect remains poorly explored, however. Preliminary analyses indicated that subordinate males create no benefits, but also no tangible costs, for resident females [81,84], but further studies of this question are warranted. Third, optimal group size in lemurs seems to be much smaller than in anthropoid primates of similar body size [85], raising the question which factors favor small numbers of females. Evidence from long-term studies indicates that female competition for reproductive opportunities is intense and ubiquitous [86]. Depending on the species, only 1 or 2 females per group may be able to reproduce successfully, and any “surplus” females either emigrate or are evicted from their natal group [87]. Thus, lemurs live in smaller groups and with more adult males than expected, given the small number of adult females.

### Social structure

Competition for mates and resources creates evolutionary incentives for establishing dominance relations in group-living species. Because the fitness of males and females is limited by different factors, they compete primarily with members of their own sex [88]. As males compete over non-divisible resources, competition is more intense and typically associated with selection for greater size, strength or weapons. The resulting physical superiority affords males with the power to dominate females, should there be conflicts of interest between the sexes. In some species, males use their physical advantage to sexually coerce females [89], but male dominance over females is generally regarded as a by-product of intrasexual selection [90].

As might be expected from the lack of male-biased sexual dimorphism, general male dominance over females has not been observed in any lemur species. Instead, adult (but not juvenile) females of some group-living species are able to evoke spontaneous submissive signals from all males and in all behavioral contexts [31,91-93]. In other species, dominance relations are weakly differentiated in general, i.e. counter-aggression is common, and individual males may consistently dominate some females, but also *vice versa,* so that there is no general effect of sex on intersexual dominance relations [94-96]. Intersexual dominance relations are not expected for solitary species, but females of some species have also been found to dominate males in experimental settings [97,98] (but see [99]). In some group-living lemurs, female intersexual dominance is backed up by larger female body size, but the extent of sexual dimorphism is generally less than 10% (Figure 1), a difference that is just considered to provide an advantage in human boxing, where weight classes change once the body weight of the heavier boxer exceeds on average 7.4 % (range 6.1 – 8.7) of the body weight of the lighter opponent (see: http://en.wikipedia.org/wiki/Weight_class_(boxing)). Thus, in group-living lemurs, general male intersexual dominance is absent and female intersexual dominance is common and only weakly related to physical superiority.

Social relationships with same-sex group-mates also distinguish group-living lemurs from most of the better-studied anthropoid primates, for which female bonding and philopatry are core aspects of sociality. While female philopatry is also the modal pattern of sex-specific dispersal in lemurs, females may also leave their natal group under two circumstances: there are better breeding prospects elsewhere [100,101] or they get forcibly evicted by other resident females [86,102,103]. Moreover, females engage in relatively more agonistic and fewer affiliative interactions than members of other dyads [104], they do not provide coalitionary support to each other [95], and they reconcile conflicts at very low rates or not at all [105-107]. Males, in contrast, can develop social bonds through regular mutual grooming, despite a lack of kinship ties [95,104,108], so that at least some male-male dyads are characterized by amicable relationships, which are only rarely found among anthropoid primates with male dispersal (see e.g.[109]). Thus, social relationships among lemur females are unusually inimical, despite close relatedness, and males exhibit more tolerance among each other than is typically found in comparable anthropoids.

### How unique is the lemur syndrome?

Some other mammals, notably some rodents and carnivores, also exhibit multiple traits that contribute to the lemur syndrome, including heightened female aggression and intersexual dominance. Before addressing hypotheses about the evolution of the lemur syndrome, a survey of some other taxa (Table 1) may therefore provide a broader perspective and guide the formulation of potential hypotheses that explain these patterns. We only briefly discuss these taxa because several recent excellent reviews have summarized the relevant evidence from proximate, ultimate and theoretical perspectives [39,110-112].

**Table 1:** Characteristics of mammalian tax a with pronounced female masculinization in multiple traits.

	Taxonomic scope	Group size	Body mass (g)	Sexual size dimorphism	Canine sexual size dimorphism	Genital masculinization	Average ASR	Female intersexual dominance	Female evictions	Singular breeding	Female bonding	Male tolerance	Modal mating system
Group-living lemurs	22 species 2 families (+ 17 subfossil)	3 - 25	1550 - 6750	0.98 (0.8-1.19)	1.06 (0.88-1.19)	moderate	0.97	widespread	common in Lemuridae	no	weak	present	Polygyny Polygynandry
Callitrichinae	@ 40 species	3 - 12	110 - 620	1.07 (0.90-1.52)	1.01(0.98-1.05)	none	1.29	absent	no;bisexual dispersal	variable	weak	high	Monogamy Polyandry
*Galea musteloides*	1 species	Not known	–	0.85 – 0.92	none	none	“male-biased”	females dominate low-ranking males	no	no	present (allonursing)	present	Polygynandry
*Heterocephalus glaber*	1 species	75	30 - 80	–	none	none	–	Queen: yes	no	yes	absent	present	Polyandry
*Crocuta crocuta*	1 species	29	59000	0.88		massive	0.55	yes	no	no	yes	present	Polygynandry
*Procavia capensis*	1 species	14	2300	1.13	None	no	0.28 – 0.13	weak	no	no	weak	low	Polygyny Polygynandry

Sexual dimorphism, genital masculinization, adult sex ratio (ASR) and female dominance are constituent variables of the lemur syndrome. References for individual entries are provided in the main text.

Tamarins and marmosets (Callitrichinae) comprise a speciose group of the New World primates. They live in small groups of 3-12 individuals, containing on average 1 to 3 adults of each sex [113]. The average adult sex ratio is generally biased in favor of males [114] but see [115]. Average sexual size dimorphism is slight (7%) and not consistently biased in favor of one sex [116], and sexual canine dimorphism is absent [117]. Female genitals are inconspicuous. Both males and females disperse from their natal group [113], a common feature of monogamous mammals. Reproduction was long thought to be limited to the dominant female [118], but more recent studies have reported the occasional presence of multiple breeding females in a range of species [114]. Thus, most callitrichine females live and mate with multiple males, which serve as carriers of their habitual twins [119]. Males are generally tolerant and groom each other [120], and intersexual dominance is not observed under natural conditions [121]. Thus, while callitrichines share some “atypical” mammalian traits with group-living lemurs, they seem to represent a highly derived social system organized around their communal breeding system.

There are no other mammalian taxa at the genus level or above that are uniformly characterized by multiple traits of the lemur syndrome. Rodents are the largest order of mammals, and the vast majority of them correspond to the modal mammalian pattern with a solitary nocturnal life style, moderate male-biased sexual dimorphism and male intersexual dominance [122]. A few interesting exceptions exist, however. In some cavies or guinea pigs (Caviinae), females are larger than males and also dominate low-ranking males [123]. *Galea musteloides* represents the best-studied example in this respect (Table 1), but their patterns of social bonding within and between the sexes differ dramatically from those in group-living lemurs. Moreover, there is substantial inter-specific variation in morphological and other social traits among cavies, indicating that traits related to female masculinization do not characterize all members of the genus, let alone the subfamily. Similarly, for some group-living species of African mole rats (Bathyergidae) in the genera *Fukomys*, *Cryptomys* and *Hetercephalus *larger female size and female dominance have been reported [124]. However, these are highly derived eusocial mammals with several castes and only one breeding female [125]. Because of their cryptic subterranean life style, little is known about their social relationships [126], but they also appear to differ from group-living lemurs in several respects (Table 1).

Carnivores also include some species with powerful females, with the spotted hyena (*Crocuta crocuta*) being the best-known example. Female spotted hyenas are larger and more aggressive than males; they dominate all immigrant males, and their genitalia feature a peniform clitoris [127-133]. However, their groups contain multiple matrilines [132], females regularly form coalitions [134] and inherit their rank [135]. Males rarely fight, they do not prevent rivals from access to females [136], and reproductive skew among them is low [137,138]. Spotted hyenas are unique in exhibiting this combination of traits because their closest relatives deviate in virtually all these traits [139]. Thus, spotted hyenas and group-living lemurs share some superficial similarities, but they differ in several respects from each other, including the taxonomic scope.

Finally, rock hyraxes (*Procavia capensis*) are the best-studied representatives of a small mammalian order (Hyracoidea) where an adult female was often found at the top of the group dominance hierarchy [140], but there are also males in every group that dominate females. Moreover, female hyraxes are smaller than males and they live in groups with strongly male-biased adult sex ratios [141].

In conclusion, the cursory summary of the traits under consideration (Table 1) indicates that group-living lemurs are unique among mammals in the particular combination of the ways these traits are shaped. Moreover, the taxonomic scope of the species exhibiting these particular trait values is much larger than in the other non-primate taxa (see also [142]), except for the callitrichines. However, compared to the callitrichines, which are a monophyletic group, this trait combination evolved twice independently among lemurs. Thus, there are indications that there is “something about lemurs” or their habitat that is important in explaining the evolution of the lemur syndrome.

### Ultimate explanations for the lemur syndrome

Previous attempts to answer the question of why the lemur syndrome has evolved have focused on its potential ultimate function. The hypotheses proposed in this context have either concentrated on lemur females and their adaptations to peculiar ecological conditions, on lemur males and the mechanisms of sexual selection, or on historical changes in ecological conditions. These hypotheses have been discussed and contrasted in detail elsewhere [32,143-147], but a brief summary of their main logic and the relevant evidence is required for the present purposes.

First, key aspects of the lemur syndrome have been related to unique ecological and environmental challenges of the Malagasy ecosystems and their consequences for female reproduction [144,146,148]. According to this ‘energy conservation hypothesis’, female dominance over males is considered an adaptive behavioral mechanism that provides adult females with feeding priority, which in turn is assumed to be beneficial or even required under the energetic stress females experience while reproducing during Madagascar's annual lean season. The key assumption of this hypothesis is that Madagascar's ecology confronts lemurs with unique challenges. Specifically, pronounced seasonality, coupled with strong climatic unpredictability, creates conditions that result in resource constraints for reproductive females, favoring adaptations that either maximize energy intake or minimize energy expenditure [32,146,149,150].

Accordingly, female dominance over males is seen as an adaptation to intersexual feeding competition, targeted aggression and female eviction represent adaptations to female competition during lactation, small group size is an adaptation to relatively low fruit productivity, and the lack of sexual dimorphism is due to energetic limitations on male body size and or female choice of compliant males [146]. This hypothesis does not offer a direct explanation for female masculinization, bonding patterns and even adult sex ratios, however. It also rests on the assumption that similar climatic conditions to the ones found today must have prevailed over much of lemurs’ evolutionary history, for which there is indeed some evidence [151]. Moreover, the same fundamental ecological challenges related to climatic unpredictability appear to override habitat characteristics across different lemur habitats [150] because the lemur syndrome is found in species inhabiting habitats ranging from dry spiny forest to coastal rain forests, but more fine-grained analyses might be instructive [152,153]. Finally, this hypothesis predicts that other Malagasy mammals, which arrived on the island independently from lemurs [154], are faced with identical ecological challenges and should therefore exhibit similar adaptive responses. The Malagasy tenrecs (Tenrecidae), mongooses (Eupleridae) and rodents (Nesomyinae) do not include group-living species, but some are pair-living [155] or have stable associations of either males or females [156]), but their social systems remain poorly known compared to those of lemurs. Patterns of sexual dimorphism are generally not pronounced [157], but in the largest carnivore (*Cryptoprocta ferox*), one class of males is about 30% larger than females [158] and adolescent females undergo transient genital masculinization [159]. Thus, currently available information does not permit general conclusions about possible similarities and differences between lemurs and other Malagasy mammals.

Second, by focusing on the absence of expected outcomes of sexual selection, one line of research has concentrated on the targets and mechanism of intra- and intersexual selection. The main hypothesis for this approach is that the intensity of male-male competition is relaxed and or overridden by female choice [143,160]. Accordingly, the pronounced seasonality of breeding observed among virtually all lemur species ought to result in a reduction of male monopolization potential, which in turn would deemphasize the importance of contest competition and might offer additional opportunities for female choice. However, recent studies of lemur mating systems using endocrine measures of individual female receptivity and genetic assessments of paternity actually revealed little evidence for strict female breeding synchrony within and between neighboring groups [74,75] (see also [161]) and strong evidence for pronounced male reproductive skew [37,54]. Pending results of similar studies in additional species, the currently available evidence does therefore not indicate that unusual intrasexual selection on lemur males can provide a sufficient and comprehensive explanation for the origin and maintenance of all components of the lemur syndrome, because male reproductive competition is fierce, ubiquitous and does not appear to follow generally different rules.

Third, the lemur syndrome has also been explained as the result of largely non-adaptive consequences of human-induced environmental changes in the last few millennia, creating an evolutionary disequilibrium between current ecological conditions and lemur traits [145]. Accordingly, the extinction of large, presumably diurnal lemur species and several large aerial and terrestrial predators following human colonization of Madagascar opened up new niches for previously nocturnal lemurs. Because primate social organization is closely linked to activity period [45], it is likely that many of these previously nocturnal species were originally pair-living, which can therefore also be expected to have been characterized by a lack of sexual dimorphism and intersexual dominance relations. As reflected by the widespread occurrence of cathemeral activity among today's group-living lemurs (or more precisely: Lemuridae) [162-165], they are still in the process of evolving into the niches occupied by diurnal group-living primates elsewhere, and 2000 years have not been sufficient to result in observable evolutionary change. Because this hypothesis was developed to explain the lemur syndrome, it does account for most of its components, and some of its specific assumptions and predictions have since been tested with mixed support [147,166-169]. It is not incompatible with the energy conservation hypotheses, however, and also highlights links between lemur ecology and behavior. Thus, current evidence about the evolution of the lemur syndrome indicates that lemur females face ecological challenges that are at least unusual and perhaps unique, compared to other primates.

### Proximate explanations for the lemur syndrome

Focusing on the proximate control of female aggression and masculinization in lemurs, several studies have explored their possible endocrinological underpinnings. As in studies of other “untypical” mammals [111], this line of research has focused on the possible role of androgenic steroid hormones in shaping female aggressive phenotypes, primarily in ringtailed lemurs (*Lemur catta*). These studies revealed that lemur pregnancies are associated with an immediate increase in androgen concentrations [170], and that female fetuses (of *E. rufifrons*) are exposed to higher androgen/estrogen ratios [171], which have the potential to exert organizational masculinization effects on developing fetuses. A comprehensive long-term study of captive *L. catta* found that female androstenedione concentrations increased during the annual breeding and birth seasons, and that female aggressive behavior towards males and other females also increased during the breeding season [172] (see also [173]).

Clearly, more endocrinological research on additional lemur species is required before the underlying assumption that circulating androgens should covary with ecologically relevant secondary sexual traits in females can be conclusively tested [174]. Nonetheless, it has been proposed that "*To the extent that hormone profiles in pregnant lemurs reflect maternal ovarian secretion and duplicate the temporal pattern seen in gestating (spotted) hyenas, a potential role for prenatal, endogenous androgens may exist in the masculinization of lemur daughters*.” [170] (p. 114). Thus, a potential proximate mechanism underlying female aggression and masculinization exists, but this line of research has not suggested a uniform ultimate reason why these traits might be adaptive. Similarly, it has been argued, based on modeling and comparative studies, that strong female dominance may arise through the self-reinforcing effects of winning and losing fights, especially in groups with larger proportion of males [175], but this line of research did not explain the phylogenetic signal in these data among primates, i.e. why the winner-loser effect has lead to strong female dominance only in lemurs. In the final section, we therefore link research on the ultimate and proximate aspects of the lemur syndrome and suggest how they may have become connected through a developmental mechanism over evolutionary times.

## A new, integrative hypothesis about the evolution of the lemur syndrome

**Figure 2:**
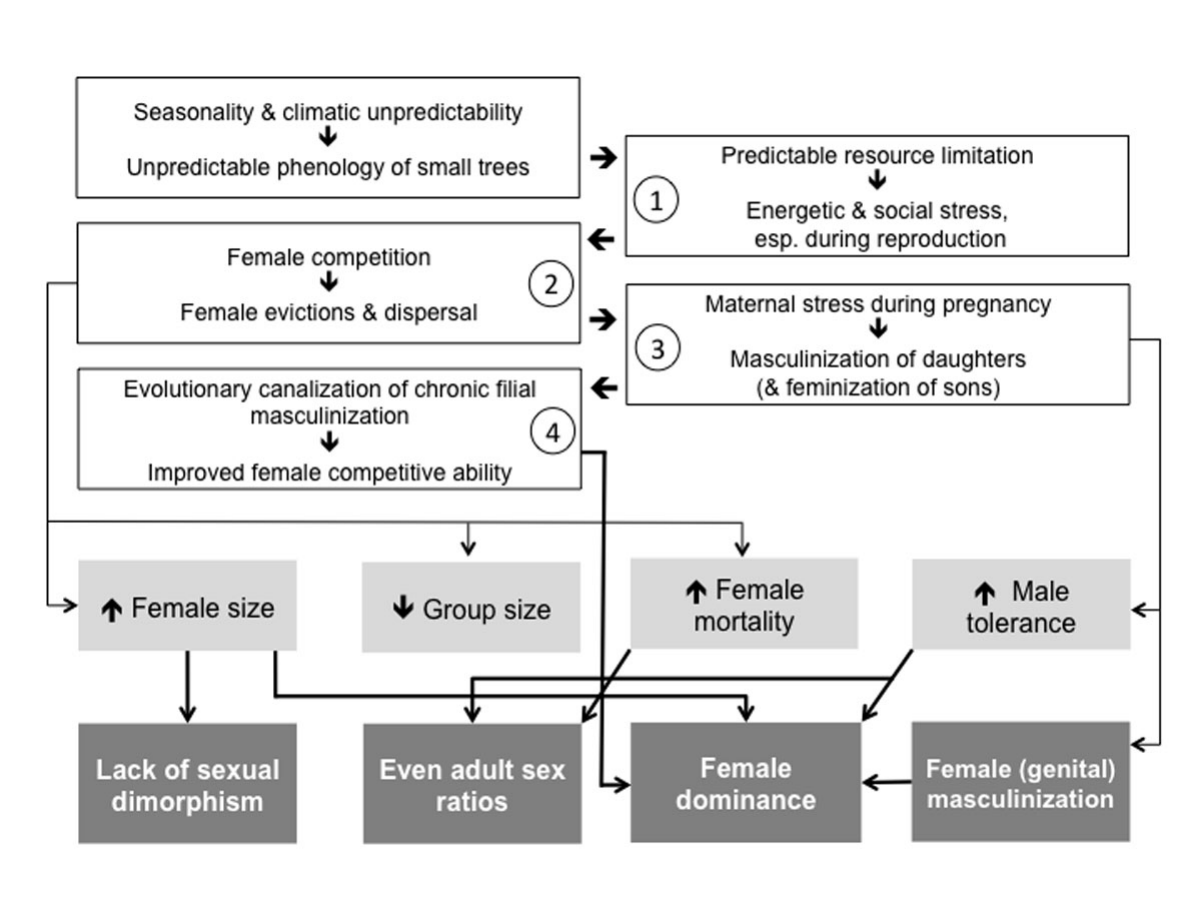
Schematic depiction of the four steps of the eco-evo-devo hypothesis to explain the evolution of the lemur syndrome. White boxes indicate logical links between the factors and processes detailed in four steps in the text. Important immediate response variables are depicted in light gray; the defining features of the lemur syndrome in dark gray.

In this final section, we combine the insights summarized above with various types of indirect evidence from studies of other taxa to develop the hypothesis that the lemur syndrome is the result of evolutionary canalization of developmental consequences of chronic maternal stress. We develop this hypothesis in four steps (Figure 2) and summarize relevant evidence, assumptions and predictions at each level.

First, we assume that lemur females are subject to significant resource limitation, especially during reproduction, creating recurrent energetic limitations. Seasonal variation in resource availability has been documented at study sites throughout the island [146,150], but seasonality in itself is not a sufficient condition because other primates and mammals in South America, Africa and Asia are exposed and adapted to similar magnitudes of climatic seasonality [176]. However, compared to these other regions harboring primates, Madagascar is characterized by more pronounced unpredictability of intra- and interannual variation in precipitation [150]. This includes humid rainforest habitats, which are characterized by high contingency, i.e. the extent to which rains fall in similar amounts in each month from year to year. Climatic unpredictability, in turn, is associated with more unpredictable periods of flowering and fruiting [150]. In addition, fruit trees in Madagascar are smaller than those in other tropical forests [177].

The combination of these effects apparently creates distinctive patterns of phenology [178,179], characterized by rather unpredictable seasonal availability of limited amounts of primate foods within the overall constraints of pronounced seasonality. Lemurs have adapted their life histories to these conditions [72,180,181], including the timing of reproduction and growth [182]. Across species, gestation and lactation of lemurs are timed such that late lactation and weaning, i.e. the periods with the greatest energy requirements, coincide with periods of presumed food abundance [146,183]. During much of their reproductive cycles, lemur females, and in particular those of larger species with absolutely longer gestation and lactation, are therefore potentially exposed to massive environmental stress [148,184] during many if not most of their lifetime reproductive events because the exact timing of food availability is poorly predictable. Thus, unpredictability is a constant of lemur ecology, i.e., energetic stress for reproducing females will arise every year (i.e. predictably), but the exact onset, strength and duration of rainfall, flowering and fruiting are unpredictable.

Some of these fundamental aspects of lemur ecology deserve additional study to better evaluate the validity of these assumptions. For example, additional data on climatic predictability should be available nowadays from many more sites around the tropics, including additional sites representing different Malagasy ecosystems, and the same is true for data on tropical tree size and long-term phenology, so that some of the pioneering studies on patterns of climatic and phenological variation can be repeated with much bigger sample sizes. In addition, there are now methods available for non-invasive measurements of energy metabolism [185], and demographic data from long-term lemur study sites can be used to investigate inter-annual covariation between climatic variation and the timing of reproductive events and postnatal growth schedules [149,186]. Furthermore, annual variation in cortisol dynamics should be studied in relation to fluctuations in environmental stressors in more detail, in additional species, and, where possible, experimentally, to determine whether any covariation represents an adaptive stress response or only an attempt to deal with recurrent stressors [187], possibly even by reducing cortisol excretion in response to chronic stress [188]. Thus, additional comparative studies are required to demonstrate the uniqueness of these aspects of lemur ecology, but the available evidence appears robust enough to assume with some confidence that lemurs are faced with some unusual and harsh ecological challenges.

Second, we postulate that group-living lemur females suffer from pronounced female competition for access to resources. Given the climatic and phenological vagaries, access to food may have limited female reproductive success on a regular basis. Females in solitary and pair-living species can alleviate these costs by spacing out [46], but females of group-living species pay a triple cost of feeding competition. First, they are larger than most solitary and pair-living species and therefore have absolutely higher energetic needs. Second, by living in groups, they suffer from feeding competition with several other adult conspecifics, including other reproductive females. Finally, the consequences of feeding competition are likely to create social stress that exacerbates the effects of the ecological stressors. This combination of effects may explain why the group-living species of the Lemuridae and Indriidae, which evolved group living independently, exhibit the lemur syndrome in a very similar manner and why some of its features are less pronounced in solitary and pair-living species.

Evidence for pronounced female competition in group-living lemurs comes from several sources. First, group size, and in particular the number of adult females, is smaller than in other primates of the same body size, and many groups of the group-living species contain only one or two adult females [85,87]. Second, in groups with more females, the probability of successful reproduction decreases significantly [86,103], and the probability of eviction (Lemuridae) or dispersal (Indriidae) increases [86,100,101]. Evictions often coincide with either the mating or birth season [86,102,189]. Finally, in female ringtailed lemurs, cortisol levels increased from an intermediate trough with both decreasing and increasing group size, presumably reflecting the effects of increasing between-group and within-group competition, respectively [190]. Female cortisol levels were also elevated in this species as a function of reproductive season, food availability and social rank [191,192]. Also, at the microhabitat level, stress levels of female collared lemurs (*Eulemur collaris*), but not of red-bellied lemurs (*Eulemur rubriventer*), increased in poorer habitats [184,193]. Nonetheless, the physiology of female stress in lemurs is surprisingly understudied, and studies on a range of species in different habitats are indicated to better characterize their response to abiotic (e.g., climate), biotic (e.g. food availability) and social stressors. Furthermore, long-term studies can contribute additional habitat- and species-specific details on the circumstances of female evictions and dispersal events. Thus, while based on studies of only a few species, the assumption of intense female competition seems justified, and selection on traits that alleviate its negative fitness consequences can be expected to be strong.

Third, based on studies in several other mammals, we postulate that maternal stress during pregnancy leads to the masculinization of daughters. It had long been noticed that prenatal maternal stress has downstream effects on offspring phenotypes [194,195], but only more recent work has identified specific effects on offspring social and sexual behavior, cognitive abilities, emotional and stress responsiveness as well as sexual differentiation and their underlying neuro-endocrine mechanisms [196-202]. Importantly, simple but elegant experiments could link biologically valid prenatal maternal stress with behavioral masculinization of daughters, including increased serum testosterone concentrations and sympathetic adrenomedullary activity, male-typical distribution of androgen receptors in the hypothalamus, and feminization of sons [197]. Also, the more aversive a mother's environment, the longer her stress response [201]. These effects are no longer regarded as pathologies, but rather as adaptive preparation for prevailing ecological and social conditions [196,198,201,203]. The observation that a relationship between maternal response to stressors, more specifically maternal cortisol levels, and offspring masculinization is also found in some fish [204] suggests that the mechanisms mediating this effect might be phylogenetically old. Recent endocrinological research on fish has furthermore suggested a possible crosstalk between the glucocorticoid and androgen pathways. Specifically, 11-β hydroxysteroid dehydrogenase (11-β HSD), an enzyme that inactivates cortisol in fish and mammals, has been hypothesized to be also implemented in the synthesis of 11-oxygenated androgens, explaining the stress-induced masculinization in some fish, essentially as a by-product of 11-β HSD properties [205].

This step of this hypothesis assumes that the same or similar prenatal endocrinological processes occur in lemurs, but the perinatal endocrinology of lemurs is poorly known. Very preliminary evidence indicates that female fetuses (in *E. rufifrons*) are exposed to much lower levels of prenatal estrogens, exposing them to higher androgen/estrogen ratios compared to male fetuses [171]. In baboons (*Papio cynocephalus*), as perhaps in other Old World primates, females pregnant with daughters also exhibit higher estrogen and testosterone metabolites than when pregnant with sons [206], but the levels in baboon females with a female fetus are only relatively lower, and not, as in the lemurs, absolutely very low. In ringtailed lemurs, pregnancy is associated with increased androstenedione and testosterone levels [170], but the sources of these steroids (maternal or fetal) remain unknown [207]. Thus, current knowledge on lemur perinatal endocrinology is insufficient to evaluate this assumption about the proximate mechanisms that may link stress with masculinization. Because the necessary invasive experiments with pregnant lemurs are ethically and legally impossible, it remains to be seen whether these assumptions of the hypothesis can be eventually tested with non-invasive methods or with comparative studies of other mammals.

Fourth, we assume that, in a final step, the recurrence of maternal stress during pregnancy and the corresponding masculinization of daughters over thousands or millions of generations have led to canalization of the effects in a way originally envisaged by Waddington [19]. Thus, masculinized daughters ought to be better prepared to compete with other females in adverse environments, so that natural selection will enhance the effects of maternal programming. Over evolutionary times, phenotypic plasticity has to be sacrificed for such canalization to occur, and adaptive canalization (i.e. benefits > costs) is indeed most likely to occur in conditions where the affected traits have direct consequences for survival or fecundity [208]. Costs of maintaining plasticity are higher in high stress environments [208], which, if persistent and predictable, further decrease the benefits of plasticity, so that natural selection is more likely to favor fixed phenotypes [209]. For example, maintaining the ability to breed year-round may not be adaptive for most lemur species and has presumably therefore been given up in favor of seasonal breeding. Finally, emerging genetic adaptations to environmental stress may have benefitted from synergistic epistasis, i.e. mutations that occur against a genetic background that has a prior history of adaptation to environmental stress can be favored [210,211]. Thus, evolutionary mechanisms to consolidate initially plastic developmental process under relevant environmental homogeneity and cost benefit ratios into a suite of stable adaptive traits do exist, and other mechanisms have been suggested for other traits subject to developmental plasticity [27]. However, despite its theoretical plausibility, it is impossible to reconstruct the details of these evolutionary processes during lemur evolution conclusively. Given a suitable model system, such as small mammal species that can be bred experimentally under laboratory conditions, however, it should be possible to test the prediction that conditions of chronic environmental and social stress will lead to stable female masculinization that persists at some point also under benign conditions.

## Conclusions

In this paper, we have summarized the current state of knowledge on an intriguing behavioral and evolutionary phenomenon in a radiation of primates. We have integrated ultimate and proximate perspectives from existing explanations of the phenomenon and proposed that an evolutionary mechanism acting upon a developmental process may link existing hypotheses for a more comprehensive explanation. This new hypothesis is difficult to test because it is about a historically completed event, but we hope that the associated assumptions and predictions about particular aspects will stimulate and guide future research on lemur behavior, ecology, physiology and development. More generally, increased appreciation of developmental processes and their potential roles as drivers of adaptive evolutionary change seems warranted.

## Authors’ contributions

PMK conceived the study and wrote the manuscript. CF contributed to discussion, data analyses and writing. Both authors have read and approved the final manuscript.

## Declarations

This publication was funded by the German Research Foundation (FOR 1232) and the Open Access Publication Fund of Bielefeld and Muenster University.
